# Canine visceral leishmaniasis: risk factors and spatial analysis in an endemic area of Northeastern Brazil

**DOI:** 10.1590/S1984-29612023029

**Published:** 2023-05-22

**Authors:** Samuel Souza Silva, Lucia Oliveira de Macedo, Jéssica Cardoso Pessoa de Oliveira, Leucio Câmara Alves, Gílcia Aparecida de Carvalho, Rafael Antonio Nascimento Ramos

**Affiliations:** 1 Laboratório de Parasitologia, Universidade Federal do Agreste de Pernambuco - UFAPE, Garanhuns, PE, Brasil; 2 Departamento de Medicina Veterinária, Universidade Federal Rural de Pernambuco - UFRPE, Recife, PE, Brasil

**Keywords:** Leishmania, epidemiology, serology, domestic dog, Leishmania, epidemiologia, sorologia, cão doméstico

## Abstract

Canine visceral leishmaniasis is a disease caused by the protozoon *Leishmania infantum*, an important agent of zoonotic concern. In this study we determined the seroprevalence, risk factors and spatial distribution of infection by *L. infantum* among dogs in the Pajeú microregion of the Sertão region of Pernambuco, Brazil. Canine serum samples (n = 247) were tested using the Dual Path Platform (DPP®) rapid screening test and ELISA/S7® confirmatory test; and risk factors were assessed through univariate analysis and logistical regression. The spatial distribution of reactive dogs was analyzed by drawing a map using QGIS. Seroprevalence of 13.7% (34/247) was detected, with cases predominating in the municipality of Tabira (26.4%; 9/34). Age above 10 years was considered to be a risk factor associated with the presence of anti-*L. infantum* antibodies. The high overall prevalence and spatial distribution of positive cases showed wide dispersion of reagents dogs in the study area. Therefore, preventive measures are needed in order to reduce the risk of infection for animals and humans.

## Resumo

Leishmaniose Visceral Canina é uma doença causada pelo protozoário *Leishmania infantum*, importante agente zoonótico. O objetivo deste estudo foi determinar a soroprevalência, avaliar os fatores de risco e a distribuição espacial da infecção por *L. infantum* em cães da microrregião do Pajeú, Sertão de Pernambuco, Brasil. Amostras de soro canino (n=247) foram testadas (“Dual Path Platform Rapid Test” - TR DPP® (triagem); ELISA/S7 (confirmatório)), e os fatores de risco avaliados por meio de análise univariada e regressão logística. A distribuição espacial dos cães reagentes foi analisada por meio de mapas confeccionados no programa QGIS. Detectou-se soroprevalência de 13,7% (34/247), com predominância dos casos no município de Tabira (26,4%; 9/34). A idade acima de 10 anos foi considerada fator de risco associado à presença de anticorpos anti-*L. infantum*. A alta prevalência e distribuição espacial dos casos positivos revelou ampla dispersão de cães reagentes na área de estudo. Portanto, medidas preventivas são necessárias a fim de reduzir o risco de infecção para os animais e humanos.

## Introduction

Visceral leishmaniasis (VL) is a disease with cosmopolitan distribution caused by the protozoon *Leishmania infantum* (Akhoundi et al., 2016). It is a matter of zoonotic concern, given that it is responsible for approximately 50,000 to 90,000 cases annually worldwide, with high occurrence rates are in Brazil, East Africa and India (WHO, 2022). The parasite is predominantly transmitted by vectors, and sandflies of the genus *Lutzomyia* are the most important vectors in the Americas (Galvis-Ovallos et al., 2021). For a long time, cases of VL were predominantly found in rural areas, but over recent years it has also been occurring in urban areas with high population density (Almeida & Werneck, 2014).

Infection by *L. infantum* can occur in several wild animal species (e.g. foxes, rodents and marsupials), as well as in domestic animals such as dogs and, less frequently, cats (Roque & Jansen, 2014; Bezerra-Santos et al., 2021; Berenguer et al., 2021). Dogs are threatened more by the disease and present a wide range of clinical signs (e.g. skin lesions, onychogryphosis, ophthalmopathies and weight loss). However, it is important to note that most infected animals remain asymptomatic, while serving as important reservoirs (Akhtardanesh et al., 2021; Peris et al., 2021). Because of the presence of asymptomatic animals in endemic regions, serological surveys are pivotal for understanding the distribution of potential reservoirs in these areas (Rondon et al., 2008; Bermudi et al., 2020).

The seroprevalence of canine visceral leishmaniasis (CVL) varies widely depending on the geographic region, level of exposure to the vectors and type of test used (Carvalho et al., 2020). For instance, in the state of Pernambuco, Brazil, prevalence rates from 2.4% (Lins et al., 2018) to 42.8% (Evaristo et al., 2020) have been detected. This serological information on dogs is useful from an epidemiological perspective, since canine cases precede human ones. In addition, early diagnosis in these animals may drive measures that should be implemented for maintaining good health status of dogs. With the aim of improving the prevention of canine infection, several risk factors have been assessed over time, and features such as living in rural areas or close to green areas, male sex and crossbreeding have been considered to be potential risk factors (Araujo et al., 2016; Evaristo et al., 2020).

It is widely known that the CVL has passed through an urbanization process in Brazil, such that the full epidemiological chain now also occurs in urban areas (Arruda et al., 2019; Batista-Santos et al., 2021). Anthropic actions (e.g. deforestation and unplanned urban growth), along with absence of basic sanitation in many regions, have certainly contributed towards worsening of this panorama (Batista-Santos et al., 2021). In this context, understanding the spatial distribution of cases and how they spread in an endemic region is pivotal to establishing barriers and avoiding dispersion of cases (Arruda et al., 2019; Evaristo et al., 2020).

Therefore, the aim of this study was to determine the seroprevalence, risk factors and spatial distribution of infection by *L. infantum* among dogs in the Pajeú microregion of the Sertão region of Pernambuco.

## Material and Methods

### Study area

**Figure 1:**
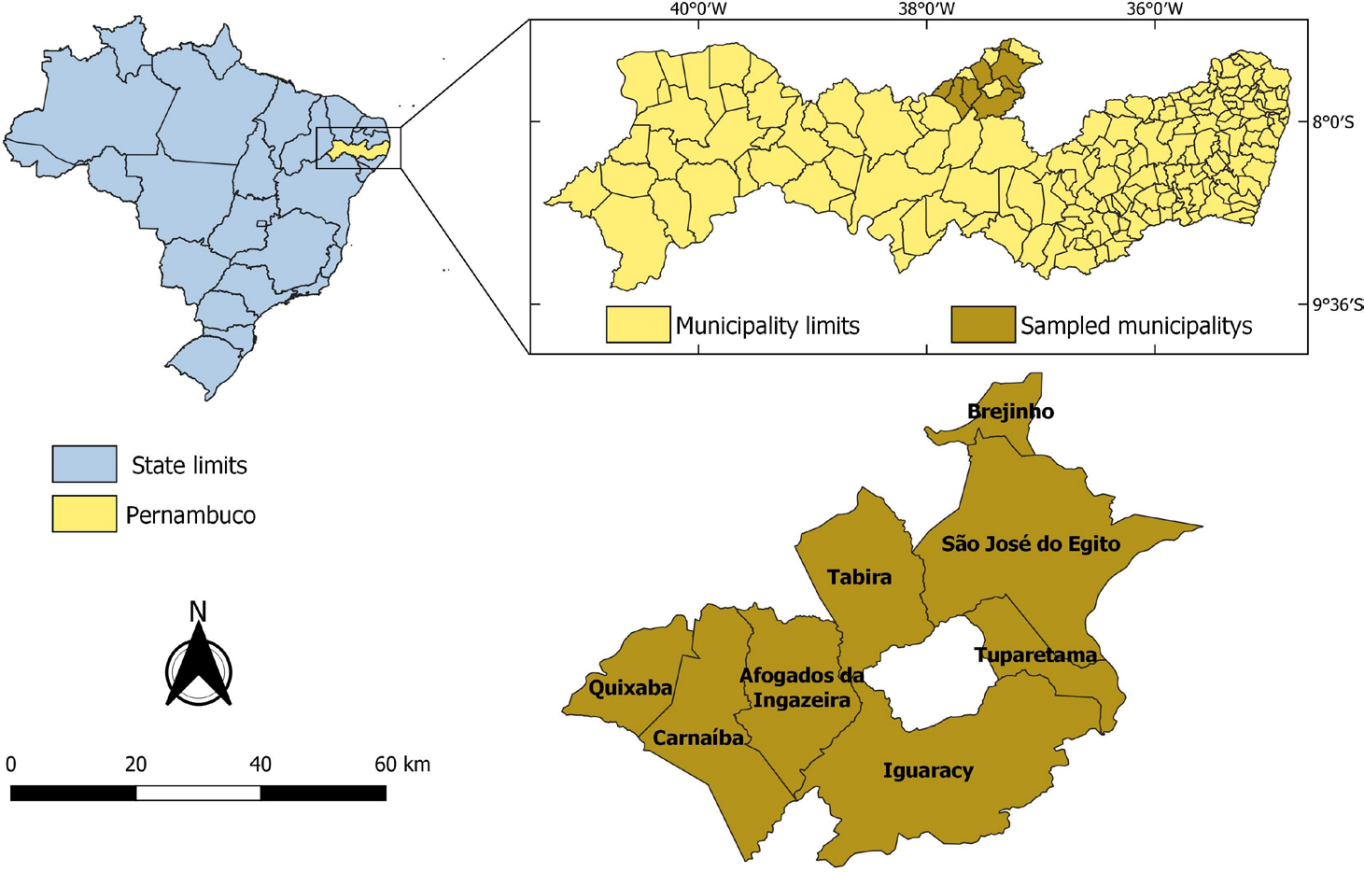
Map of Brazil indicating the state of Pernambuco and municipalities where animals were sampled.

This study was conducted in the Pajeú microregion of the Sertão, in the state of Pernambuco, northeastern Brazil. Eight municipalities from which human VL cases were notified from 2009 to 2019 were included in the study: Afogados da Ingazeira (07°45'03” S and 37°38'21” W), Brejinho (07°20'58” S and 37°17'10” W), Carnaíba (07°48'19” S and 37°47'38” W), Iguaracy (07°50'07” S and 37°30'55” W), Quixaba (07°43'13” S and 37°50'54” W), São José do Egito (07°28'44” S and 37°16'28” W), Tabira (07°35'27” S and 37°32'22” W) and Tuparetama (07°36'08” S and 37°18'41” W) (Figure 1). The study area is characterized by a hot and dry tropical climate. It forms part of the caatinga biome, has stretches of hyper-xerophilous forest and has an average temperature ranging from 17 to 36 °C. The rainy season runs from November to July, with an average annual precipitation that varies from 5 to 118 millimeters (IBGE, 2022).

### Animals, sampling and serological examination

The minimum sample size required (n = 246) was calculated considering the estimated canine population of the region (n = 35,129). This requirement followed the proportion established in a previous study (Canatto et al., 2012). A margin of error of 5% and a confidence interval of 95% were taken into account (Thrusfield, 2004). The collection points in each municipality were randomly determined (Reis, 2003).

Blood samples were obtained from animals of both sexes, different breeds and aged between 6 months and 13 years-old. A physical examination was performed on each animal and clinical signs suggestive of infection by *L. infantum* were recorded in individual clinical charts. Additionally, an epidemiological questionnaire was applied to each owner to obtain data for analysis of risk factors.

All samples were screened using the Dual Path Platform (DPP^®^) rapid test (Bio-Manguinhos/FIOCRUZ, Rio de Janeiro, Brazil), which is a qualitative test that detects antibodies anti-*Leishmania* IgG. Afterwards, reactive samples were analyzed through a quantitative ELISA/S7^®^ test (Biogene, Recife, Brazil) and antibody concentrations were measured in a spectrophotometer at absorbance of 450 nm. All tests were performed in accordance with the manufacturers' recommendations, and animals were considered reactive when they were found to be positive in both techniques.

### Data analysis

The data were analyzed using descriptive statistics to obtain absolute and relative frequencies. Subsequently, the G test was used to assess the seropositivity of dogs in each municipality, as well as in both rural and urban areas of these municipalities. Risk factors were calculated using univariate analysis for the variable of interest and by means of logistic regression in which the serology result was taken to be the dependent variable. The significance level was taken to be 5% in both analyses. The G test and risk factor analyses were conducted using the BioEstat software, version 5.3 (Instituto Mamirauá, Brazil), and the Epi-Info^TM^ 7.2.2.6 software, respectively.

### Spatial analysis

The geographic coordinates of each collection point (each animal’s home) were obtained through the global positioning system (GPS). Maps were created using the QGIS 3.22.10 software, in which georeferenced data were inserted in continuous cartographic base maps (Shapefiles, version 2017), which are available from the database of the Brazilian Institute for Geography and Statistics (IBGE, 2022).

A thematic map and a Kernel density map were created to indicate the distribution of cases and clusters of positive cases, respectively. An influence radius of 3 km with pixels size 100 were used to generate a good visualization of plotted points in the raster layer. In addition, considering the mean dispersion of vectors (250 m), a buffer zone was established surrounding positive and negative cases, to demonstrate risk areas of potential transmission.

## Results

The overall seroprevalence obtained was 13.7% (34/247). The highest number of positive animals (26.4%; 9/34) was in the municipality of Tabira. Non-reactive animals were observed in Afogados da Ingazeira, Brejinho, Iguaracy and Tuparetama. The detailed results from the serological analysis, according to the municipality and collection area (urban or rural), are presented in Table 1.

**Table 1:** Detection of anti-*Leishmania* (*Leishmania*) *infantum* antibodies among dogs in rural and urban areas of the Pajeú microregion.

**Municipality**	**Urban area (UA)**			**Rural area (RA)**			**Prevalence by municipality**
	**DPP**	**ELISA**	**DPP+ELISA**	**DPP**	**ELISA**	**DPP+ELISA**	
Afogados da Ingazeira	0/16	-	0/16	-	-	-	0/16
Brejinho	1/12 (8.33%)	0/1	0/12	-	-	-	0/12
Carnaíba	5/6 (83.33%)	4/5 (80%)	4/6 (66.67%)	4/14 (28.57%)	1/4 (25%)	1/14 (7.14%)	5/20 (25%)
Iguaracy	0/3	-	0/3	-	-	-	0/3
Quixaba	1/7 (14.29%)	1/1 (100%)	1/7 (14.26%)	38/137 (27.74%)	17/38 (44.74%)	17/137 (12.41%)	18/144 (12.5%)
São José do Egito	3/9 (33.33%)	2/3 (66.66%)	2/9 (22.22%)	-	-	-	2/9 (22.22%)
Tabira	1/1 (100%)	1/1 (100%)	1/1 (100%)	10/33 (30.30%)	8/10 (80%)	8/33 (24.24%)	9/34 (26.47%)
Tuparetama	0/6	-	0/6	-	-	0/3	0/9

A total of 13.9% (26/187) of the animals living in rural areas were reactive, while among those living in urban areas the seroprevalence was 13.3% (8/60). It is important to note that in the municipalities of Tabira and Quixaba, the reactive animals were predominantly found in rural areas, rather than urban areas (G test = 10.2792; p = 0.0059).

Among all the animals evaluated, 53% (131/247) presented at least one clinical sign. Among the seroreactive animals, 61.8% (21/34) presented clinical signs suggestive of infection by *L. infantum*. Lymphadenomegaly (38.2%; 13/34) and alopecia (23.5%; 8/34) were the signs most commonly observed (Table 2).

**Table 2:** Clinical signs observed among all sampled/seropositive dogs, and the frequency of signs among seropositive animals.

**Clinical signs**	**Dogs with clinical signs**		**Frequency (%)**
	**Total**	**Seropositive**	
Alopecia	32	8	23.5
Paronychia	5	1	2.9
Onychogryphosis	35	7	20.6
Signs eyes	14	3	8.8
Gastrointestinal signs	43	5	14.7
Disorder micturition	3	0	0
Lymphadenomegaly	62	13	38.2
Anorexia	1	1	2.9
Hyporexia	14	4	11.8

From the univariate analysis, the only risk factor identified was the animals’ age. In particular, those older than 10 years of age were more prone to become infected (*OR* = 4.94; p = 0.029). Table 3 shows the analysis on risk factors associated with the presence of anti-*L-infantum* antibodies.

**Table 3:** Univariate analysis on risk factors associated with the presence of anti-*Leishmania* (*Leishmania*) *infantum* antibodies in dogs in the Pajeú microregion.

**Variables**		**N**	**Positives**	**Univariate analysis**	***p*-value**
			**n (%)**	**OR (CI 95%)**	
Hunting	Yes	69	9 (13.0)	0.91 (0.35 - 2.18)	0.509
	Not	178	25 (14.0)		
Housing	Residence	57	7 (12.3)	0.84 (0.29 - 2.14)	0.451
	Peridomicile	190	27 (14.2)		
Area	Rural	187	26 (13.9)	1.04 (0.42 - 2.85)	0.551
	Urban	60	8 (13.3)		
Ectoparasites	Yes	103	12 (11.7)	0.73 (0.31 - 1.63)	0.266
	Not	144	22 (15.3)		
Sex	Male	175	24 (13.7)	0.98 (0.42 - 2.45)	0.558
	Female	72	10 (13.9)		
Age	<5 years	174	19 (10.9)		
	≥5 to 10 years	61	10 (16.4)	1.59 (0.62 - 3.89)	0.184
	≥10 years	10	5 (50.0)	4.94 (0.95 - 26.22)	0.029*
Pelage	Short	210	32 (15.2)	
	Average	34	2 (5.9)	0.34 (0.03 - 1.48)	0.110
	Long	3	0 (0.0)	0.00 (0.00 - 70.07)	0.842

N: total samples; n: total positive and negative samples; OR: odds ratio; CI: confidence interval.

* p <0.05, significant association.

**Figure 2:**
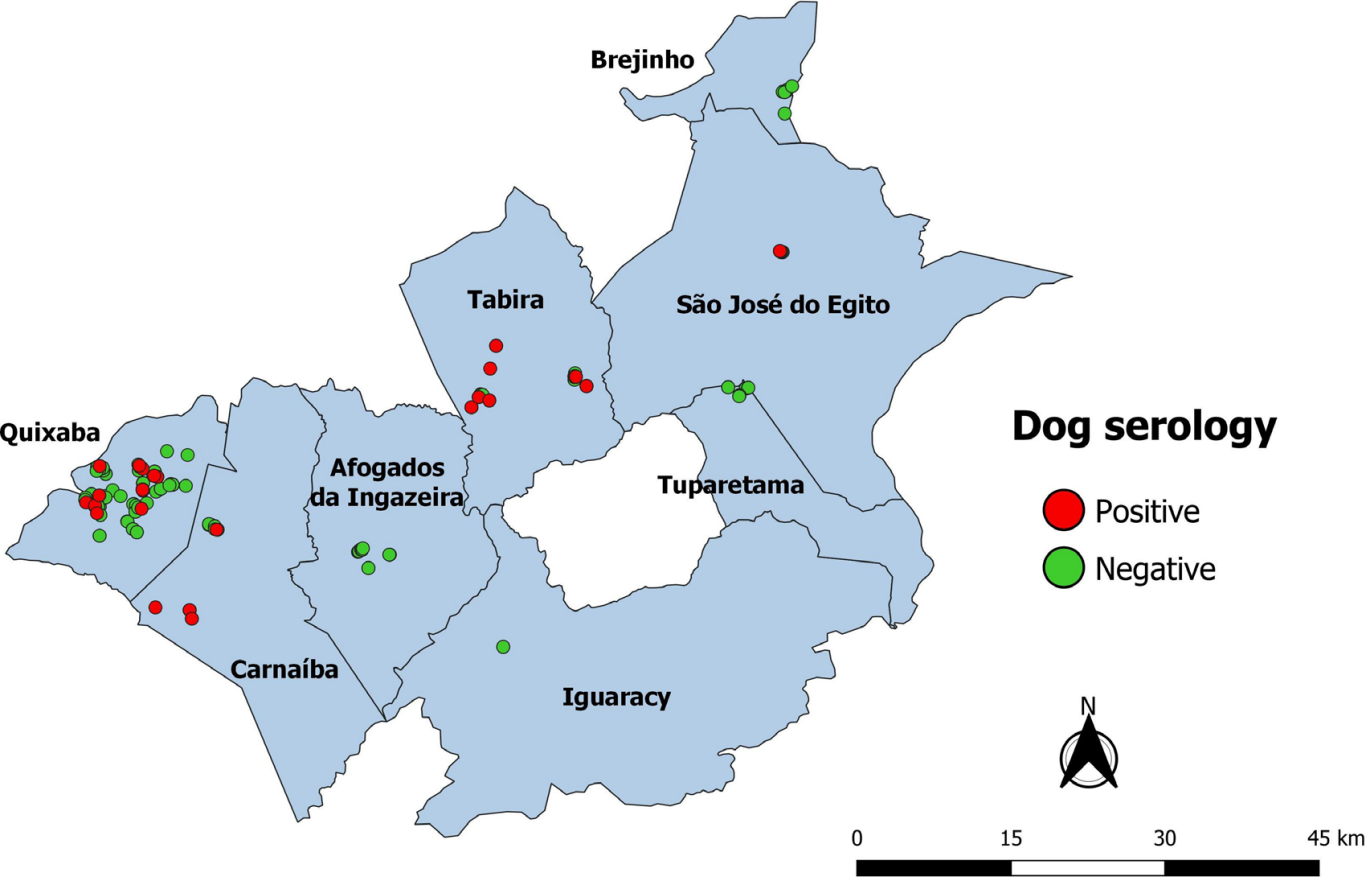
Map indicating the distribution of reactive and non-reactive animals regarding the presence of anti-*L. infantum* antibodies.

The spatial analysis revealed that reactive dogs were widely distributed in the study area (Figure 2).

**Figure 3:**
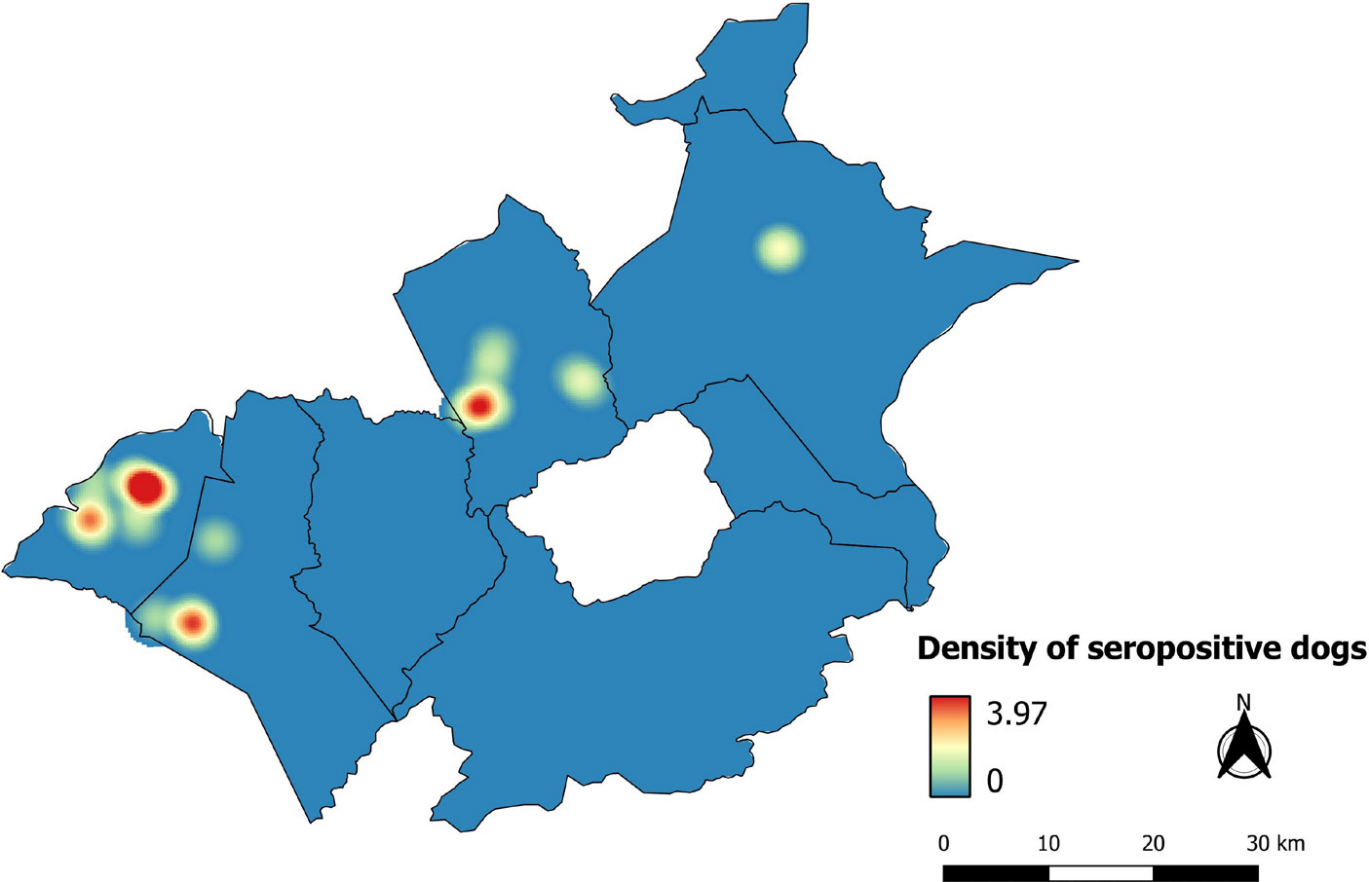
Kernel density map demonstrating clusters of animals that were reactive regarding the presence of anti-*L. infantum* antibodies.

The Kernel density map revealed clusters of positive cases in the urban areas of three municipalities (Carnaíba, Quixaba and Tabira). Moreover, the rural area of Quixaba also demonstrated a cluster of reactive cases (Figure 3).

**Figure 4:**
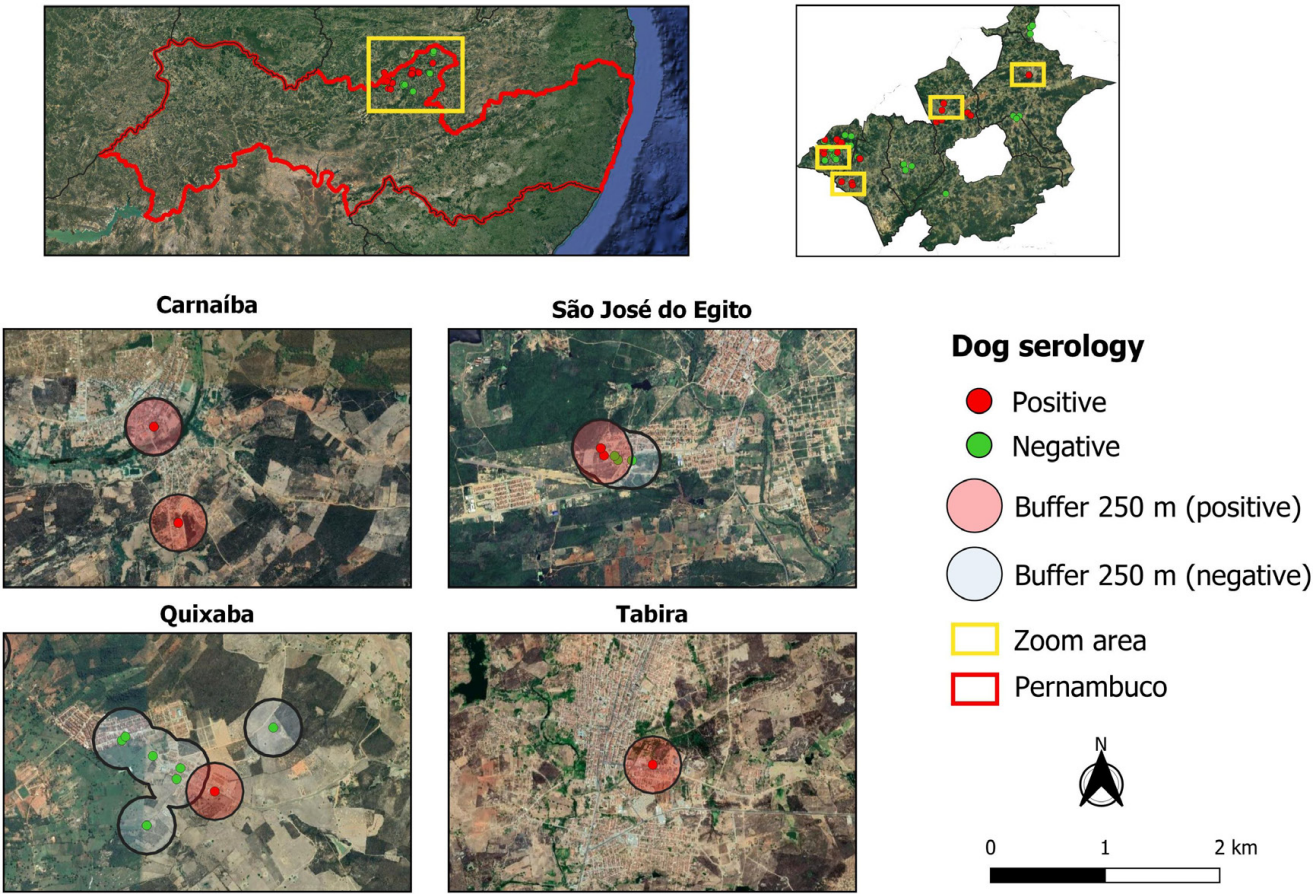
Map indicating the buffer zones (250 m) surrounding the locations of reactive and non-reactive animals regarding the presence of anti-*L. infantum* antibodies.

Confluence of the buffer zone between positive and negative cases was observed. It is also important to note that some overlapping between vegetated areas and the buffer zone of positive cases also occurred (Figure 4).

## Discussion

In this study, the presence of anti-*L. infantum* antibodies in dogs was assessed. It was demonstrated that age (> 10 years) was a risk factor for occurrence of reactive cases, which were widely distributed across the study area.

The overall seroprevalence (13.7%) indicates that *Leishmania* parasites were circulating in the population assessed, thus following a trend that had already been observed in other regions of the state of Pernambuco (Dantas-Torres et al., 2006; Araujo et al., 2016; Lins et al., 2018; Macedo et al., 2022). Recently, in a study carried out in the Sertão region of Pernambuco, a mean prevalence of 13.9% was reported (Evaristo et al., 2021), similar to what was found in the present study.

It is known that this region presents favorable climatic conditions for vector development (Macedo et al., 2008). Additionally, a wide variety of wild hosts (rodents, canids and marsupials) that are epidemiologically important in relation to leishmaniasis have been observed in this area. Nonetheless, in four municipalities (Afogados da Ingazeira, Brejinho, Iguaracy and Tuparetama), no reactive animals were observed. However, this result should be interpreted with caution, due to the low number of samples (n = 40), which may have influenced the final outcome. The low number of samples in some municipalities is considered a limitation of this study, which difficult a more reliable analysis. It is known that the study area presents environmental features (e.g. climate, relative humidity, vegetation and wild host population) that are similar to the conditions required for vector development and occurrence of cases.

No difference between rural areas (13.9%; 26/187) and urban areas (13.3%; 8/60) was observed (G test = 0.0095; p = 0.9223). Even if CVL cases have occurred predominantly in rural areas, the urbanization of the disease over recent years has been responsible for occurrences of cases in areas with high population density (Araujo et al., 2016). Urbanization of CVL is a negative consequence of anthropic actions (deforestation, disorderly urban growth and lack of basic sanitation), which have favored movement of wild reservoirs closer to urban areas, as well as establishment of vector populations (Barbosa et al., 2010; Pimentel et al., 2015). Furthermore, studies conducted since the urbanization of VL was first recognized have suggested that there is a positive correlation between the presence of infected dogs and VL outbreaks among humans (Mello et al., 2014; Sales et al., 2019).

Clinically, the most common signs found in our study were lymphadenomegaly (38.2%; 13/34) and alopecia (23.5%; 8/34), which are frequently observed in dogs with VL (Akhtardanesh et al., 2021; Peris et al., 2021). It is important to highlight that 38.2% (9/34) of the seroreactive animals were asymptomatic. From an epidemiological point of view, these animals are very important, since they may act as sources of infection while remaining unperceived because of their clinical status. Therefore, it is necessary to carry out serological surveys for early detection of these cases and for preventive measures to be adopted (Rondon et al., 2008; Bermudi et al., 2020). The univariate analysis revealed that age (> 10 years) was considered to be the sole risk factor (*OR* = 0.029). Despite of important, this data should be interpreted with caution since only ten animals with more than 10-year-old were sampled. Even though, it is known that the longer exposure to vectors and the immunological weakness observed among elderly patients may contributed to this outcome (Araujo et al., 2016). In fact, adult dogs remain outside for long periods, which increases the chance of contact with vectors and natural exposition to *Leishmania* parasites (Selim et al., 2021).

The Kernel map showed clusters of CVL cases in five municipalities, thus demonstrating that this type of mapping is an important tool that can identify specific zones where preventive measures are needed. Active surveillance should be implemented in these areas in order to reduce the risk of transmission and occurrence of human and animal cases (Araujo et al., 2016; Evaristo et al., 2020). The buffer zone (250 m) around the locations of positive dogs showed overlaps with forested areas, thus indicating a bridge between these areas. From an epidemiological perspective, this is very important because it may indicate close contact between vectors and domestic and wild reservoirs. Additionally, it reflects the indiscriminate growth of cities, which has been an important factor in the urbanization of the disease (Batista-Santos et al., 2021).

The data presented here are very important, especially because the distribution area of CVL has expanded in several regions, with domestic dogs as the main source of infection in these places (Oliveira et al., 2021; Silva et al., 2021; Veloso et al., 2021). Although the lack of collection of sandflies can be considered a limitation of this study, it is known that the study area presents favorable environmental conditions for their development (Macedo et al., 2008). Furthermore, during the samplings, a great diversity of livestock (e.g. horses, pigs and ruminants) and some synanthropic animals (rodents and marsupials) were observed in peridomestic areas. This highlights the wide variety of food sources for vectors, as well as the presence of reservoirs of *L. infantum*, which contribute to occurrence of the disease.

VL continues to cause deaths of humans and dogs in endemic regions of Brazil (Sousa et al., 2018; Oliveira et al., 2021). The measures that should be taken to control VL are widely known, but the divergent ways in which these preventive actions are applied, along with the poor infrastructure of many municipalities, make controlling this disease a great public health challenge. Awareness about CVL control in Brazil has improved over recent years (Belo et al., 2017), but in many regions this control is still based on isolated measures focusing especially on dogs (Alves et al., 2018). The best way to control neglected diseases is through the One Health approach, in which the conditions of humans, animals and the environment in which these hosts live are considered together (Costa et al., 2020; Silva et al., 2021; Brasil, 2014).

In summary, the findings from this study demonstrated the presence of anti- *L. infantum* antibodies in dogs and indicated that age (> 10 years) was a risk factor. Preventive measures (use of repellent products, control of vectors and environmental management) are needed in order to reduce the risk of infection among animals, thus mitigating the potential impact on local public health.
